# Case Report: Beyond the stomach: the role of preoperative imaging in assessing regurgitation risk in patients with esophageal dilatation: a report of two cases

**DOI:** 10.3389/fmed.2026.1933635

**Published:** 2026-10-09

**Authors:** Kecheng Huang, Rongpeng Pi, Jieyu Fang

**Affiliations:** 1 Department of Anesthesiology, Guangxi Hospital Division of the First Affiliated Hospital, Sun Yat-sen University, Nanning, Guangxi, China; 2 Department of Anesthesiology, The First Affiliated Hospital, Sun Yat-sen University, Guangzhou, Guangdong, China

**Keywords:** achalasia, computed tomography, endoscopic decompression, esophageal carcinoma, rapid sequence induction, regurgitation

## Abstract

**Background:**

Perioperative regurgitation of gastric or esophageal contents remains a severe complication during anesthesia. While standard fasting protocols and rapid sequence induction (RSI) are routinely employed, esophageal dilatation creates a hidden reservoir for retained food and secretions that conventional methods often fail to mitigate. Furthermore, standard gastric ultrasound lacks the field of view to detect mid-to-proximal esophageal pooling.

**Case presentation:**

We present two parallel cases emphasizing this critical challenge. Case 1 involves a 68-year-old male with esophageal carcinoma who experienced massive regurgitation of white foam during RSI, despite five days of preoperative fasting. Airway security was ultimately achieved after intensified suctioning. Case 2 describes a 42-year-old female with achalasia and marked esophageal dilatation. Informed by preoperative computed tomography (CT) and barium swallow imaging, she was safely managed using targeted pre-anesthetic conscious endoscopic suctioning, leading to an uneventful induction and peroral endoscopic myotomy (POEM).

**Conclusion:**

These cases highlight the limitations of conventional fasting in managing proximal pooling. They underscore the diagnostic value of preoperative cross-sectional imaging, such as CT, when clinically indicated. Active pre-induction decompression, specifically through tailored endoscopic evacuation, is a valuable adjunctive strategy for preventing perioperative regurgitation in selected patients with megaesophagus.

## 1 Introduction

Perioperative regurgitation of esophageal contents represents a significant risk to patient safety, potentially leading to severe pulmonary aspiration (1). Esophageal dilatation (megaesophagus) can result from various underlying etiologies, including motility disorders (e.g., achalasia), mechanical obstruction (e.g., esophageal carcinoma), and chronic inflammatory conditions (2, 3). Patients with a dilated esophagus are at a uniquely high risk of regurgitation and aspiration during anesthesia induction (4).

Generally, extended fasting and rapid sequence induction (RSI) are recommended to avoid regurgitation and aspiration (5). However, these standard protocols do not provide an absolute guarantee, particularly when a distal obstruction causes massive proximal pooling. While endoscopy, barium esophagography, and high-resolution manometry are primary modalities for diagnosing esophageal motility disorders, CT plays a distinct and critical role in defining the anatomical extent of dilatation, characterizing retained contents, and evaluating extrinsic or malignant causes. In this report, we present two cases that highlight the diagnostic advantage of preoperative imaging in identifying these hidden risks and discuss individualized pre-induction management strategies.

## 2 Case presentation

### 2.1 Case one: esophageal carcinoma

A 68-year-old male presented with a history of dysphagia, vomiting, hematemesis, and regurgitation. He had been diagnosed with esophageal squamous cell carcinoma and was scheduled to undergo gastrostomy tube placement for enteral nutritional support.

Preoperative computed tomography (CT) revealed an obstructing esophageal carcinoma with significant proximal esophageal dilatation containing retained fluid, blood, and air mixed with food debris. The maximum dilation was located in the mid-esophagus, measuring 4.7 cm × 6.1 cm. (Figure 1).

**Figure 1:**
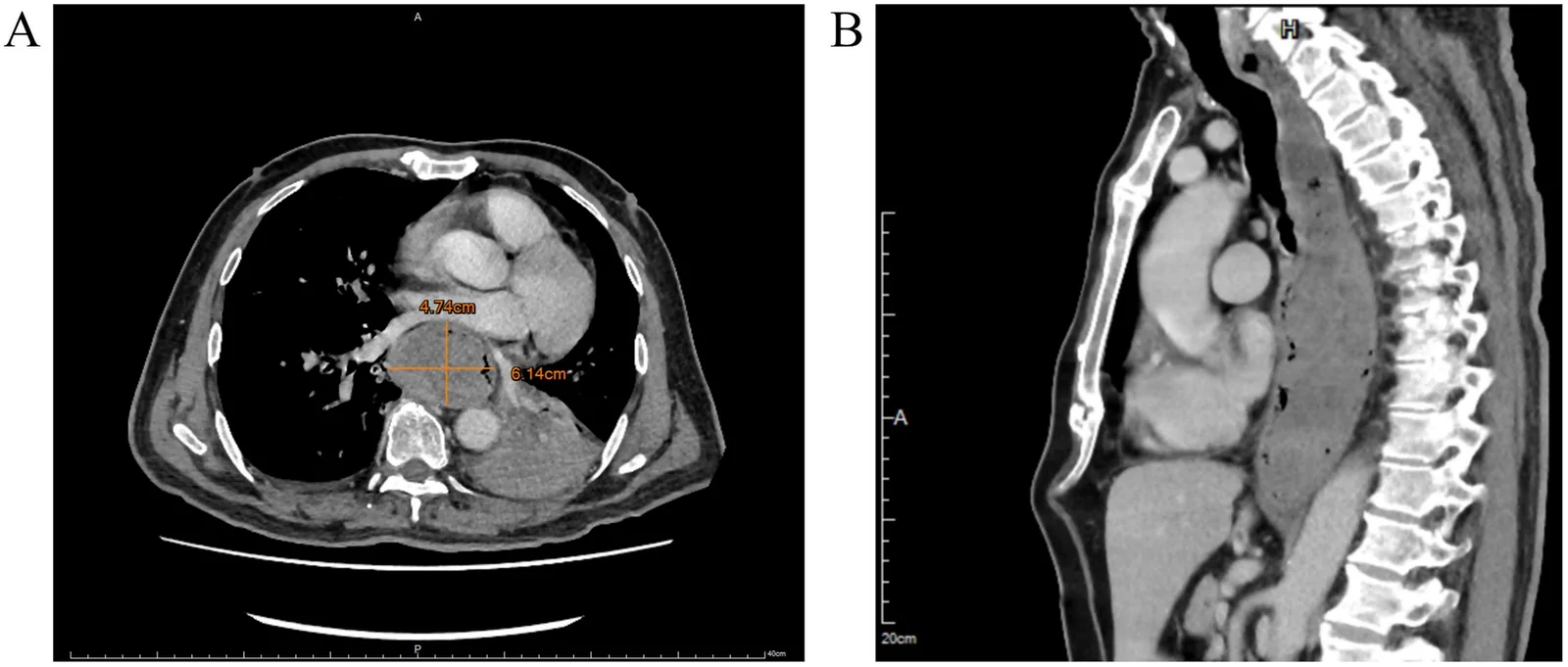
Esophageal dilatation secondary to esophageal carcinoma. Computed tomography (CT) imaging demonstrated an esophageal carcinoma resulting in obstruction, with associated proximal esophageal dilatation containing retained fluid and blood. The presence of air, admixed with food debris, was also noted. **(A&B)** The maximum dilation is located in the mid-esophagus, with a size of 4.7 cm × 6.1 cm..

The patient was fasted for 5 days preoperatively with parenteral nutrition support. Recognizing the high risk of regurgitation, the anesthesiologist prepared for RSI. The patient entered the operating room without a gastric tube in place. He was positioned supine, and after preoxygenation for 10 min, RSI was initiated using propofol, rocuronium, and sufentanil. Cricoid pressure was immediately applied. Immediately following the loss of consciousness, a massive amount of white foam emerged from the esophagus into the oral cavity (Figure 2). During the initial intubation attempt using a videolaryngoscope, the sheer volume of foam completely obscured the lens. Following prompt and intensified suctioning, a good laryngoscopic view was obtained, and endotracheal intubation was successfully achieved on the first attempt.

**Figure 2:**
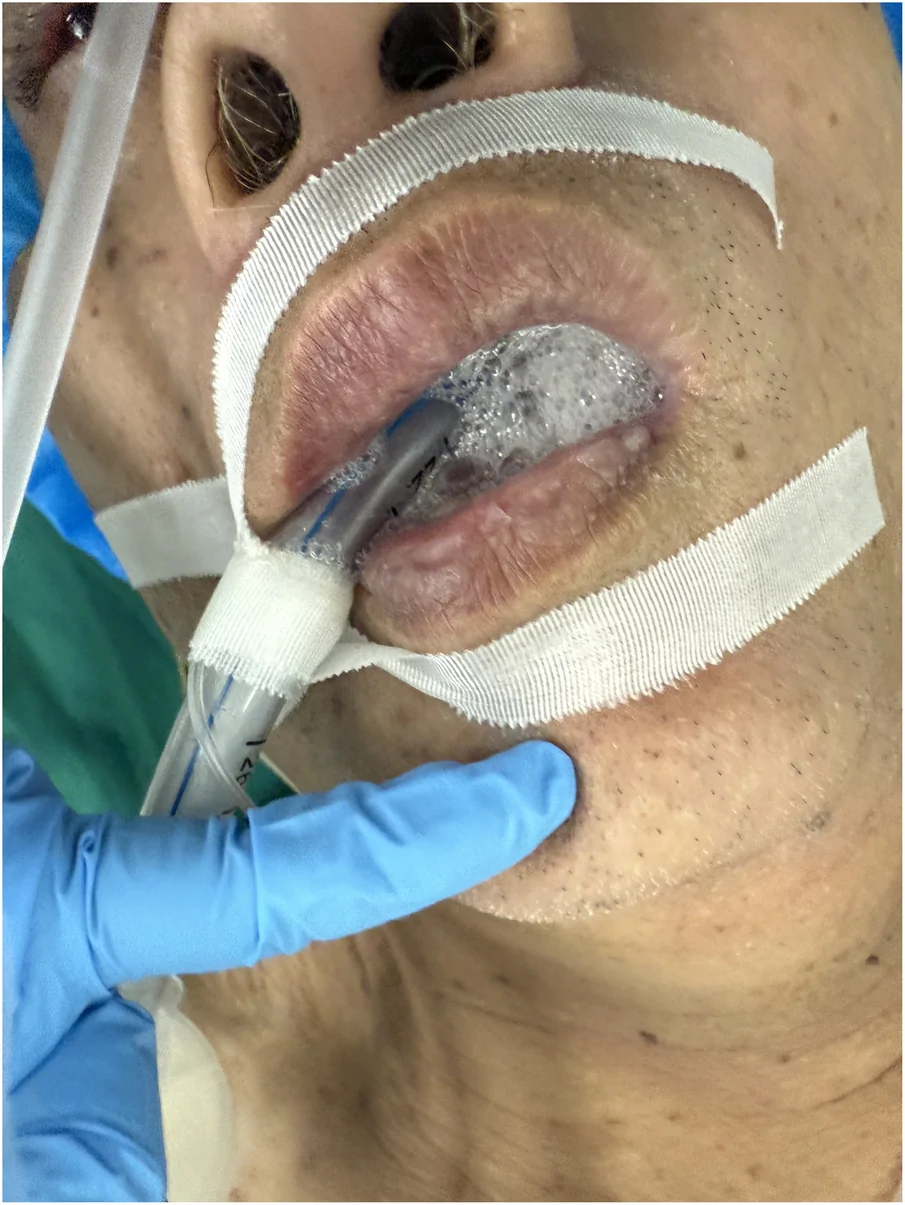
Esophageal regurgitation. A large amount of white foam emerged from esophagus into the mouth.

Notably, white foam continued to pour from the esophagus even after intubation. However, no foam or gastric fluid was aspirated from the endotracheal tube itself. Intraoperatively, the patient received volume-controlled positive pressure ventilation, maintained stable hemodynamics, and kept normal oxygenation throughout the surgery. Postoperative chest radiography revealed no obvious abnormalities, and the patient was closely monitored for 72 h without any significant adverse respiratory events.

### 2.2 Case two: achalasia

A 42-year-old female presented with dysphagia and vomiting and was diagnosed with achalasia. She was scheduled to undergo peroral endoscopic myotomy (POEM) under general anesthesia.

A preoperative barium swallow study demonstrated characteristic findings of achalasia cardia, revealing significant dilatation of the thoracic esophagus with a smooth, tapered narrowing at the gastroesophageal junction resembling a “bird's beak” morphology (Figure 3A). Supine CT imaging confirmed marked dilatation of the esophagus with significant intraluminal retention of food debris and associated air-fluid levels, consistent with stasis secondary to functional obstruction (Figure 3B).

**Figure 3:**
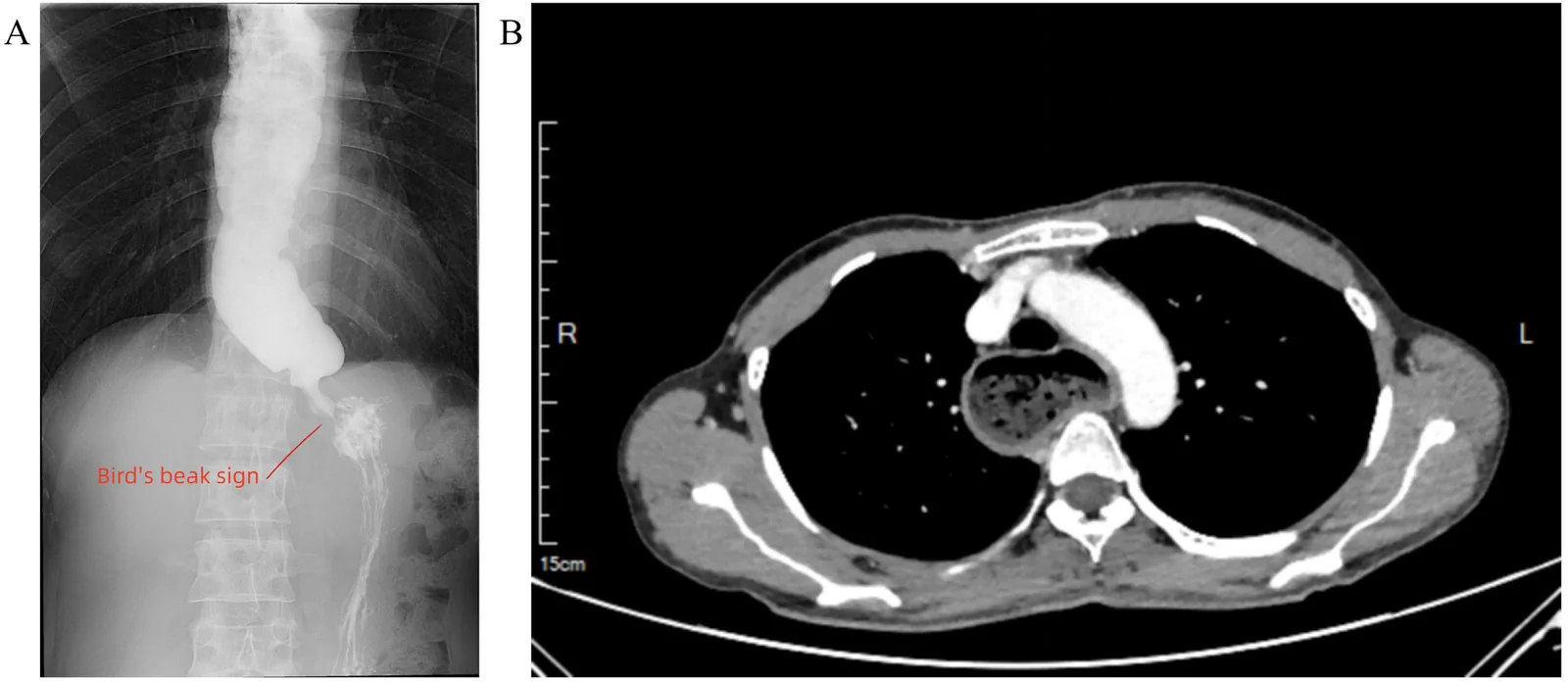
**(A)** “Bird’s beak” sign in achalasia. Barium swallow showing smooth, tapered narrowing at the gastroesophageal junction (“bird’s beak” sign) in achalasia. **(B)** Esophageal dilatation. Supine CT shows esophageal dilatation with food residue, air-fluid levels.

Based on these imaging findings, a tailored approach was adopted. The patient underwent a 24-hour fasting period. Ten minutes prior to anesthesia induction, the patient was placed in the left lateral decubitus position. Following topical oropharyngeal local anesthesia with lidocaine gel, and without the administration of any intravenous sedation, a gastroenterologist performed conscious endoscopic suctioning to evacuate residual material from the dilated esophagus. A quantity of bile-like fluid was successfully evacuated. Following this active decompression, the patient was repositioned, and routine induction was performed using propofol, sufentanil, and rocuronium. Endotracheal intubation was achieved uneventfully without any evidence of regurgitation. The POEM procedure was completed without complications.

## 3 Discussion

The parallel presentation of these two cases demonstrates the hidden dangers of esophageal dilatation and the necessity of individualized anesthetic planning. Standard fasting guidelines designed for healthy elective surgical patients do not apply to patients with structural or functional esophageal obstruction. As observed in Case 1, even after five days of strict fasting and parenteral nutrition, significant amounts of saliva and swallowed air can accumulate in the esophageal reservoir. The massive white foam regurgitated during induction highlights that prolonged fasting alone is insufficient to empty a mechanically or functionally obstructed esophagus.

While RSI is the gold standard for full-stomach patients, it is not foolproof. In Case 1, the sheer volume of regurgitant obscured the videolaryngoscope, creating a “cannot intubate” crisis that was only resolved through aggressive suctioning. If a nasogastric or orogastric tube had been placed pre-induction, it might have reduced the intraluminal pressure and the volume of regurgitated fluid, even if it could not pass the malignant stricture.

Conversely, Case 2 illustrates a highly effective, individualized management pathway. By utilizing CT and barium swallow imaging to definitively identify the anatomical risk, the multidisciplinary team opted for pre-anesthetic conscious endoscopic suctioning. Crucially, preoperative cross-sectional imaging plays a valuable role when clinically indicated. Beyond merely demonstrating esophageal dilatation and retained contents, CT helps define the level and nature of the obstruction, which is essential for planning subsequent endoscopic or surgical management. Consistent with this, recent literature highlights the utility of thoracic CT in the diagnostic evaluation of esophageal obstructions, emphasizing that early diagnosis and timely intervention are critical for reducing complications (6).

Although point-of-care gastric ultrasound is highly effective at indicating reflux risk due to gastric retention, it fundamentally lacks the field of view to rule out risk caused by proximal esophageal pooling. Therefore, for patients with suspected megaesophagus, interventions must be tailored. Conscious endoscopic clearance before sedation, as performed in Case 2, represents a secure strategy to mitigate regurgitation risk. However, endoscopic intervention itself carries procedural and patient discomfort risks. Therefore, this strategy should not be viewed as a universal algorithm; rather, management must be individualized based on the underlying etiology, urgency of the procedure, local gastroenterological expertise, and the patient's clinical characteristics and tolerance.

### 3.1 Limitations

We acknowledge that this report is limited by its inclusion of only two cases, which feature entirely different underlying pathophysiological mechanisms (malignant mechanical obstruction versus functional achalasia). Further large-scale observational studies are needed to establish standardized risk-stratification criteria for patients with varying etiologies of megaesophagus.

## 4 Conclusion

Esophageal dilatation poses a severe, under-recognized risk for perioperative regurgitation that conventional fasting cannot adequately manage (1). Cross-sectional imaging, when available and clinically indicated, is highly valuable for identifying retained esophageal contents and informing anesthetic planning. While RSI remains a cornerstone technique, strategies such as targeted endoscopic evacuation or nasogastric suctioning prior to anesthetic induction should be carefully considered as part of an individualized approach to securing the airway in high-risk patients.

## Data Availability

The original contributions presented in the study are included in the article/Supplementary Material, further inquiries can be directed to the corresponding author.
